# A Case Report of Capsular Contracture Immediately Following COVID-19 Vaccination

**DOI:** 10.1093/asjof/ojab021

**Published:** 2021-05-22

**Authors:** Richard J Restifo

## Abstract

Capsular contracture is fundamentally an immunological/inflammatory response to the implant, treating it as a foreign body in need of exclusion from the immune system. The capsule surrounding the implant is populated by a rich variety of immunologically active cells such as macrophages, T lymphocytes, and myofibroblasts. Vaccination in general and the COVID-19 vaccine in particular result in specific and nonspecific activation of the immune system, including those immune cells in proximity to the implant. This phenomenon has been previously demonstrated in delayed inflammatory reactions to previously implanted hyaluronic acid fillers following COVID-19 vaccination. This report is what is believed to be the first case of the rapid development of severe ipsilateral capsular contracture in the immediate aftermath of the second dose of the BNT162b2 (Pfizer) vaccine.

Capsular contracture is a multifactorial process with immunologic and inflammatory components. The end result of this process is a fibrotic foreign body reaction surrounding the implant, which is the body’s attempt to isolate the implant from the immune system. The cellular composition of the capsule includes macrophages, lymphocytes, fibroblasts, and contractile myofibroblasts.^1^ The capsule/implant contact zone demonstrates a multilayered accumulation of immunologically active cells, including activated CD4^+^ T cells.^2^ These cells in and around the capsule can produce a variety of profibrotic cytokines, including transforming growth factor-beta 1 (TGFβ1) and several interleukins.^3^ The reasons why the inflammatory/immunologic process abates in most patients, but in other patients continues long after the initial surgical insult, are incompletely understood and are what drives most capsular contracture research. Certain factors, such as hematoma, seroma, or subclinical infection/biofilms, are considered to be triggers to the continued immunologic/inflammatory response that leads to contracture.^4,5^ Strategies to prevent or treat capsular contracture target these processes and include meticulous surgical technique, steroids, leukotriene inhibitors, antibiotic-coated mesh as well as a variety of other anti-inflammatory modalities.^6-8^

The COVID-19 pandemic has drastically altered many facets of everyday life, to say nothing of the public health hazard that has resulted in over 3 million deaths worldwide at the time of this writing. Fortunately, several effective vaccines have made it to the market and the rate of vaccination is accelerating in many countries. An effective vaccine should elicit both an antibody response and a T-cell–mediated response,^9^ and the BNT162b2 (Pfizer, New York, NY) vaccine has been shown to cause a rise in antigen-specific neutralizing antibodies as well as in CD8^+^ and CD4^+^ T cells,^10^ which presumably underlie its 95% efficacy in terms of preventing primary COVID-19 infection. This stimulation of an immune response by the vaccine is not without collateral effects, which fortunately have been largely limited to mild local (pain, swelling), regional (lymphadenopathy), and systemic (headache, fevers, chills, and myalgias) reactions. There has also been a handful of delayed inflammatory reactions to previously implanted hyaluronic acid fillers, which although requiring treatment were not life-threatening. Most of these were following the mRNA-1273 (Moderna, Cambridge, MA) vaccine, but there has been one reported case where a patient experienced infraorbital swelling at the site of a tear trough injection (two and a half years previously) following the second dose of the Pfizer vaccine.^11^

This manuscript presents a case in which a patient with silicone implants placed approximately 6 months previously developed a sudden and severe capsular contracture of one breast following the second Pfizer vaccine dose. To the author’s knowledge, this is the first report of this type.

## CASE REPORT

**Figure 1.**
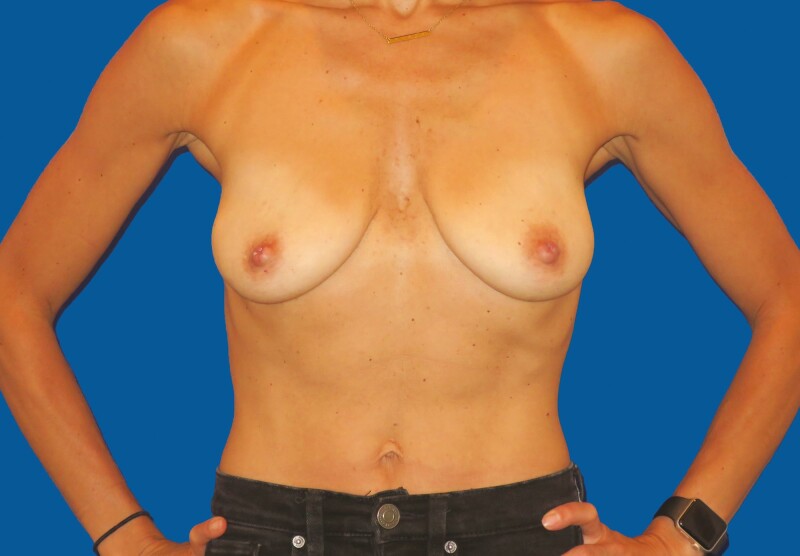
A 34-year-old healthy woman presented for augmentation/mastopexy. Preoperative AP view.

**Figure 2.**
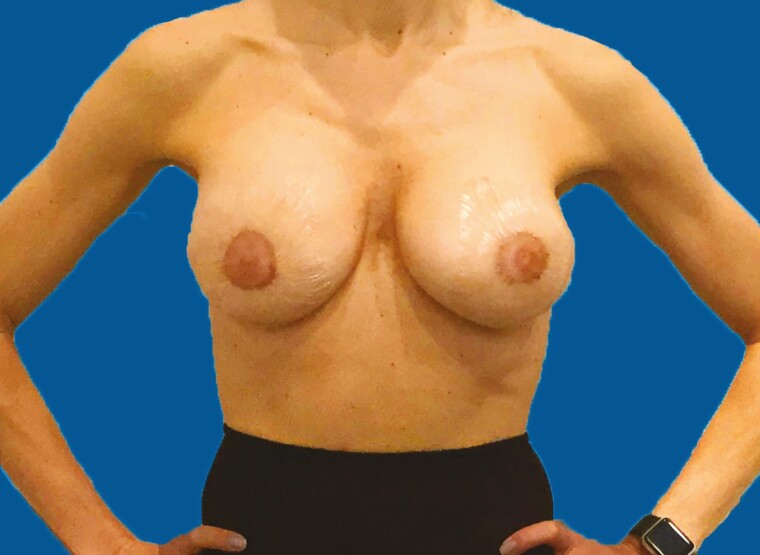
Six weeks’ postoperative AP view. Implants are soft and moveable (Baker I).

A completely healthy gravida 3, para 3 woman was seen in consultation for postpartum mammary involution and ptosis (Figure 1). Subsequently, she underwent augmentation/periareolar mastopexy with a subpectoral 440 cc smooth-walled implant. Preoperative intravenous cefazolin (1 g) was given. The implant pocket was irrigated with triple antibiotic solution (1 g cefazolin, 50,000 U of bacitracin, 80 mg gentamicin) as well as with povidone-iodine solution. Poly-4-hydroxybutyrate (GalaFLEX, Galatea Surgical, Inc, Lexington, MA) mesh reinforcement was used inside the implant pocket; this product is routinely used by the author for augmentation/mastopexies to support both the parenchyma and the implant position. Tegaderm nipple shields, routinely used by the author for augmentations, were not used in this case due to the need to transpose the nipple-areolar complex. The implant was placed with an insertion funnel through a separate inframammary incision. Postoperatively she did well, and at 6 weeks, postoperative photographs demonstrate good implant position (Figure 2), and at 10 weeks, postop was noted to have good implant position with soft and movable implants (Baker I) bilaterally.

**Figure 3.**
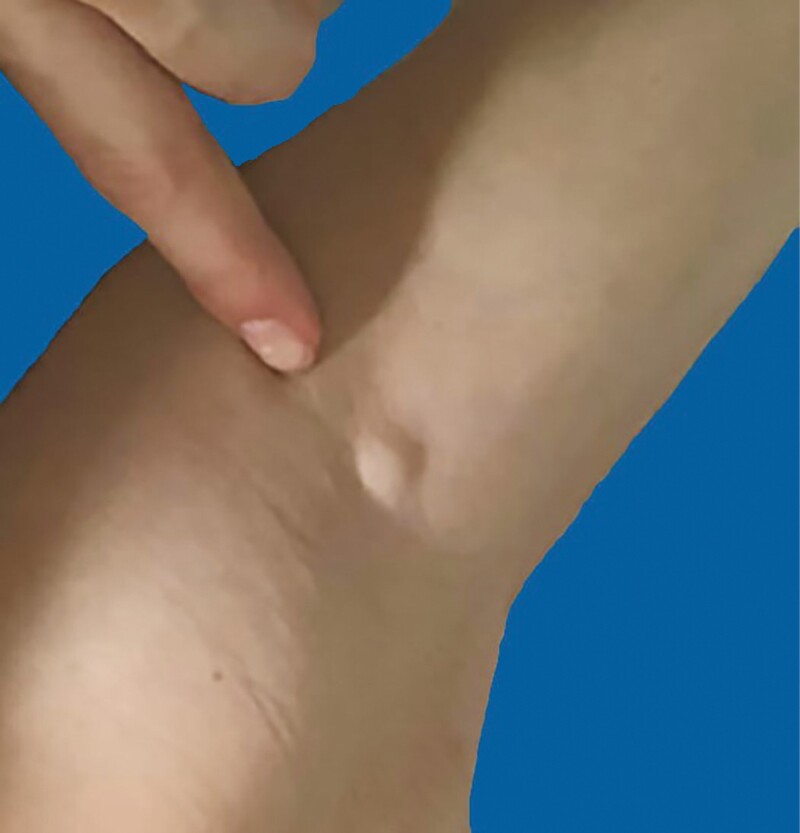
Six and one-half months’ postoperative AP view/6 days after the second dose of Pfizer vaccine. Enlarged left axillary lymph node.

**Figure 4.**
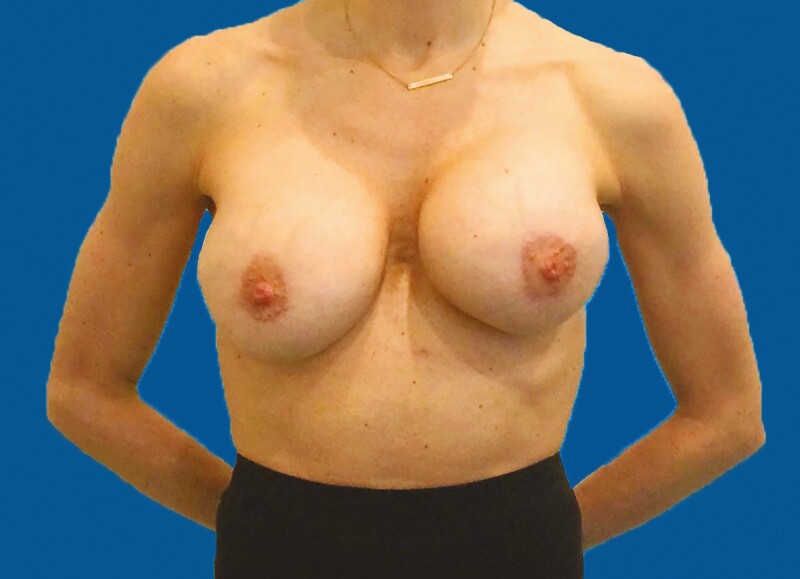
Seven months’ postoperative AP view/20 days after the second dose of Pfizer vaccine. Left breast is hard and painful (Baker IV).

Five months postoperatively she had the first dose of the Pfizer vaccine and 21 days later had the second dose; both injections were placed in the left shoulder. Six days after the second dose, she noted an enlarged lymph node in her left axilla (Figure 3). Thirteen days after the second dose, she reported that her left breast was firm, swollen, and “tight”. Soon after this, she was started on montelukast. The situation progressed until 20 days after the second dose, she was examined and seen to have a painful, distorted Baker IV capsular contracture (Figure 4). Because of the severity of her symptoms, she underwent early revisional surgery with capsulectomy and implant exchange. Intraoperatively she was found to have an intact implant and a dense fibrotic capsule containing 37 cc of a thin reddish-brown fluid but no hematoma or organized blood clot.

## DISCUSSION

Although capsular contracture is typically thought of as a process that takes place over weeks to months,^12^ there is experimental evidence that provides a mechanism for capsular contracture evolving over only a few days, as seems to have occurred in this case. For example, tissue cultures obtained from the contracted capsules of women with silicone implants demonstrated the ability to induce a rapid upregulation of the genes for TGFβ1, interleukin 8 as well as other inflammatory and profibrotic cytokines in vitro, which in turn, were thought to be responsible for the stimulation of contracture of fibroblast-populated collagen lattices over a 48- to 120-hour period.^13^ Another study also using tissue media derived from the capsules of women with implants showed enhanced T-cell proliferation and increased cytokine levels as compared to peripheral blood.^3^ These studies further support the concept of an immunologically mediated and rapid process for capsular contracture; the cellular composition of the capsule and the surrounding tissue seems poised to respond to an immunological stimulus.

It is possible that the timing of the capsular contracture vis-à-vis the vaccine was a mere coincidence. The contracture occurred during the typical time frame for contracture (most are within the first year^14^), and it occurred following an operation (augmentation/mastopexy) that has a high contracture rate.^15^ In fact, the presence of a clearly hemorrhagic collection within the capsule raises an alternative hypothesis, that a small delayed hematoma was the cause of the contracture. There are just over 30 case reports in the literature of hematomas occurring at least 6 months following cosmetic breast augmentation.^16^ Many of these had instigating events, such as trauma or physical strain or a recent iatrogenic coagulopathy, and many occurred in cases with aggressively textured implants, where the separation of the implant from the adherent capsule was thought to be the reason for the bleeding. This patient had none of these possible factors in her history. She denied trauma, was not engaged in implant displacement exercises or any kind of weightlifting, and her coagulation tests and platelet counts were normal. In delayed hematomas without a discernible etiology, the so-called called spontaneous cases, several authors suggest a variety of nonspecific inflammatory conditions as possible causes.^17-19^ Along these lines, the inflammatory response seen in a delayed hypersensitivity reaction (as in the aforementioned post-vaccine reactions to hyaluronic acid fillers) can induce angiogenesis and increased vascular permeability within days in animal models.^20^ Furthermore, the complex inflammatory and angiogenesis cascades share some of the same mediators, for example, TGFβ1 and interleukin 8^20-22^ and can be activated in concert.^23^ Therefore, the finding of a small amount of a bloody fluid in the capsule does not preclude an inflammatory, vaccine-induced mechanism for capsular contracture. Additionally, with the tight temporal sequence of events—the second dose of the vaccine, then lymph node enlargement, then a discernible tightening that rapidly progressed to a Baker IV contracture—it seems hard to completely exculpate the vaccination as a possible cause, by one mechanism or another.

It is also possible that the placement of the poly-4-hydroxybutyrate mesh in direct opposition to the implant contributed to the contracture, although the limited evidence that is available seems to suggest, if anything, that poly-4-hydroxybutyrate is more preventative than causative when it comes to capsular contracture.^24^ The ipsilaterality of the contracture to the vaccine site is also interesting; one might suspect that a systemic response to a vaccine would demonstrate no such side preference, although in this case there was an ipsilateral regional reaction with the enlarged lymph node.

It would be premature to propose any protocol based upon a single case report. The preemptive use of zafirlukast has been suggested, for augmented patients getting the vaccine who experience any tightening, on at least 1 plastic surgical practice website and this makes sense from a mechanistic point of view. However, as a blanket recommendation for all augmented patients getting the vaccine, this would seem to treat many patients unnecessarily with a drug that is not entirely benign. Furthermore, even though zafirlukast acts upon a somewhat distal part of the immunological/inflammatory cascade (suppression of myofibroblasts), *any* interference with the body’s immune response immediately after the vaccine might seem to be unwise and counterproductive to the entire point of the vaccine. Certainly at this point patients with implants should not avoid COVID-19 vaccination, consistent with The Aesthetic Society president’s recommendation that patients with previous hyaluronic acid fillers be vaccinated without hesitation.

## CONCLUSIONS

This report details the case of a woman who developed severe capsular contracture that may have been precipitated by COVID-19 vaccination. As the pace of vaccination accelerates, more capsular contracture cases may appear which may necessitate the formulation of specific treatment guidelines.
